# Adjuvant laser meridian massage in men with opioid use disorder on methadone maintenance treatment: Protocol for a case-controlled study

**DOI:** 10.1097/MD.0000000000017319

**Published:** 2019-09-27

**Authors:** Wen-Long Hu, Meng-Chang Tsai, Chun-En Kuo, Chun-Ting Liu, Szu-Ying Wu, Tzu-Chan Wu, Yu-Chiang Hung

**Affiliations:** a Department of Chinese Medicine, Kaohsiung Chang Gung Memorial Hospital and Chang Gung University College of Medicine; b Fooyin University College of Nursing; c Kaohsiung Medical University College of Medicine; d Department of Psychiatry, Kaohsiung Chang Gung Memorial Hospital and Chang Gung University College of Medicine, Kaohsiung; e Department of Nursing, Meiho University, Pingtung; f Department of Sports Medicine, Kaohsiung Medical University, Kaohsiung; g School of Chinese Medicine for Post Baccalaureate I-Shou University, Kaohsiung, Taiwan.

**Keywords:** heroin addiction, laser meridian massage, methadone maintenance treatment, opioid use disorder, traditional Chinese medicine

## Abstract

**Background:**

Heroin addiction remains a significant public health problem worldwide, and relapse to heroin use following cessation of agonist maintenance treatment is common. The problems associated with use of opioid agonists mean that non-opioid therapies need to be developed to ameliorate acute and protracted opioid withdrawal syndromes.

**Methods:**

Fifteen men with opioid use disorder on methadone maintenance treatment have been enrolled from an addiction treatment center as an experimental group in this case-controlled study. This group is receiving laser meridian massage on the back, including the Bladder meridian and Governor Vessel, 3 times weekly for 4 weeks. An age-matched control group that does not receive laser meridian massage has also been enrolled. Urinary morphine levels are being checked before and after 2 and 4 weeks of treatment. Subjects are requested to self-report their number of episodes or days of heroin use and 0 to 10-point visual analogue scale scores for heroin craving/refusal to use heroin during the previous week before and after 2 and 4 weeks of treatment. Quality of life will be reported using the Short Form-12v2 before and after 4 weeks of treatment. Pulse diagnosis will be recorded and heart rate variability calculated after one single treatment session. The baseline patient characteristics will be compared between the experimental and control groups using the independent *t* test and Chi-square test. Data are compared between the 2 groups using repeated-measures analysis of variance, generalized estimating equations, and the paired *t* test.

**Objective:**

To investigate the effect of adjuvant laser meridian massage in men with opioid use disorder on methadone maintenance treatment.

**Trial registration:**

ClinicalTrials.gov NCT04003077.

## 1 Introduction

Opioid use disorder (OUD), which most commonly manifests as heroin addiction, remains a significant public health problem worldwide. Clinically, OUD is characterized by physical dependence, as evidenced by tolerance and withdrawal, and by psychological symptoms, including drug cravings, depression, anxiety, and inability to control use of heroin.^[1]^ Methadone maintenance treatment is the most widespread and extensively researched treatment for heroin addiction. Methadone maintenance has been shown to reduce the frequency of opioid use, reduce mortality, reduce the transmission of human immunodeficiency virus and viral hepatitis, improve employment prospects, and reduce the frequency of criminal behavior. Many of these positive effects are strongly associated with higher daily methadone dose and with increasing duration of treatment.^[2]^ However, relapse to heroin use following cessation of agonist maintenance treatment is common. Research is lacking on when, who, and how to withdraw from opioid agonist maintenance treatment. The problems associated with use of opioid agonists have made it necessary to develop non-opioid therapies to ameliorate the symptoms of acute and protracted opioid withdrawal.^[3,4]^

Acupuncture is becoming a popular complementary and alternative treatment in Western countries and is growing in popularity worldwide.^[5,6]^ Acupuncture is based on traditional Chinese medicine (TCM) theory and was developed according to the principle that human bodily functions are controlled by the “meridian” and “Qi and blood” systems. There are 365 designated acupuncture points located along 14 meridians that can be used to stimulate, balance, and harmonize the yin and yang by relieving blockages in the flow of Qi.^[7]^ This method of healing has been used to promote homeostasis of the body's organs.

Stimulation of acupoints has been used in heroin addicts since 1972. Dr Wen reported that acupuncture combined with electrical stimulation at 4 body points (IL4, SI3, EH4, and TB9) and 2 ear points (brainstem and shenmen) relieved the withdrawal syndrome in heroin addicts.^[8]^ In 1985, Dr M. Smith, the head of the US National Acupuncture Detoxification Association, developed a protocol that consisted of insertion of 5 needles without electrical stimulation bilaterally into the outer ear or auricle at points referred to as sympathetic, shenmen, kidney, lung, and liver. The National Acupuncture Detoxification Association reported that the 5-point auricular acupuncture protocol relieves the withdrawal syndrome, prevents symptoms of craving, and increases the likelihood of a patient participating in a long-term treatment program.^[9]^ Electroacupuncture at the Bei-Shu acupoints has been reported to attenuate the prolonged early-stage withdrawal symptoms following heroin detoxification and to suppress anxiety.^[10]^ Governor Vessel acupoints are commonly used in the treatment of heroin addiction.^[11]^ Laser acupuncture is a noninvasive technique that involves stimulation of traditional acupoints with low-intensity, non-thermal laser irradiation. Laser acupuncture integrates the positive effects of both acupuncture and low-level laser therapy.^[12]^ Laser meridian massage is the application of laser acupuncture that involves stimulation along the meridian with low-intensity, non-thermal laser irradiation. The aim of this study is to investigate the effect of adjuvant laser meridian massage in men with OUD on methadone maintenance treatment according to TCM theory, which advocates treatment of the meridians to ameliorate symptoms of opioid withdrawal.

## 2 Methods

### 2.1 Ethics approval

The study was approved by our institutional human ethics committee (Chang Gung Medical Foundation Institutional Review Board, approval number 201801823A3). The protocol was registered with ClinicalTrials.gov (identifier NCT04003077). Written informed consent will be obtained from all study participants. Personal information about potential and enrolled participants will be collected, shared, and maintained in an independent closet in order to protect confidentiality before, during, and after the trial.

### 2.2 Study design

**Figure 1:**
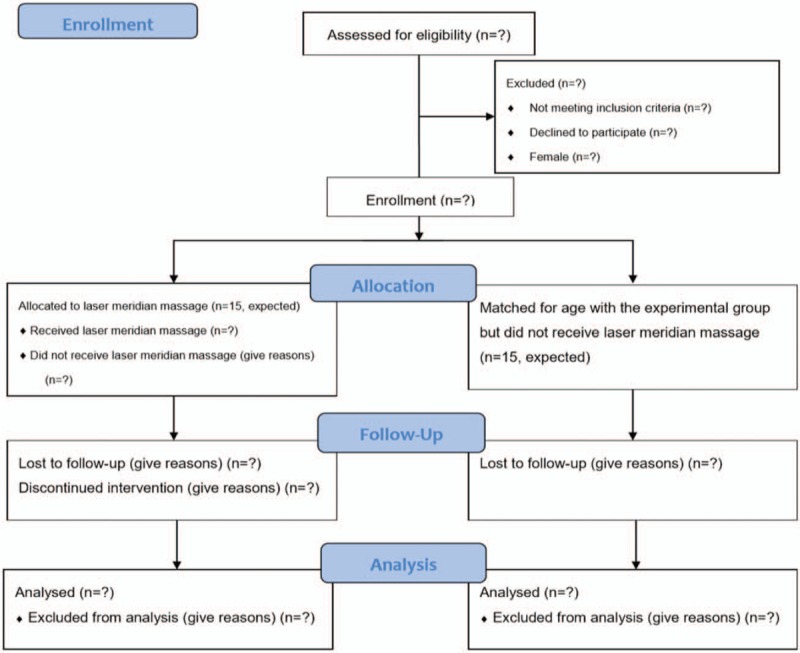
Flowchart showing movement of subjects through the study.

This case-controlled study is being performed in the Department of Psychiatry and Chinese Medicine at Kaohsiung Chang Gung Memorial Hospital. The study was started in February 2019 and will continue until February 2020. Subjects recruited from our institution's addiction treatment center are being allocated to the experimental group (laser meridian massage plus methadone maintenance treatment, n = 15, expected). The study participants will receive 12 sessions of laser meridian massage three times weekly for 4 weeks. An age-matched control group of subjects who do not receive laser meridian massage (methadone maintenance treatment only, n = 15, expected) will also be enrolled. The study design is shown in Figure 1.

### 2.3 Participants

A diagnosis of OUD is confirmed using the diagnostic criteria of the Diagnostic and Statistical Manual of Mental Disorders, Fifth Edition. Male subjects aged 20 to 70 years with OUD who have received methadone maintenance treatment for at least 1 month and provided informed consent are being recruited. Psychiatrists assess each prospective participant's eligibility to be enrolled in the study. Subjects with a critical illness or human immunodeficiency virus infection, those on antidepressant or antipsychotic medication, those who have taken Chinese herbs or received acupuncture treatment during the previous 30 days, those who are unsuitable for recruitment in the opinion of the attending physician, and those who are unwilling to provide informed consent are excluded.

### 2.4 Sample size

A sample size of 28 was calculated to be needed based on 2-way repeated-measures analysis of variance with a medium effect size of 0.25, a significance level (α) of 0.05, and a desired power (1−β) of 0.80.^[13]^ Anticipating a 7% dropout rate, a total of 30 study participants will need to be recruited.

### 2.5 Interventions

**Figure 2:**
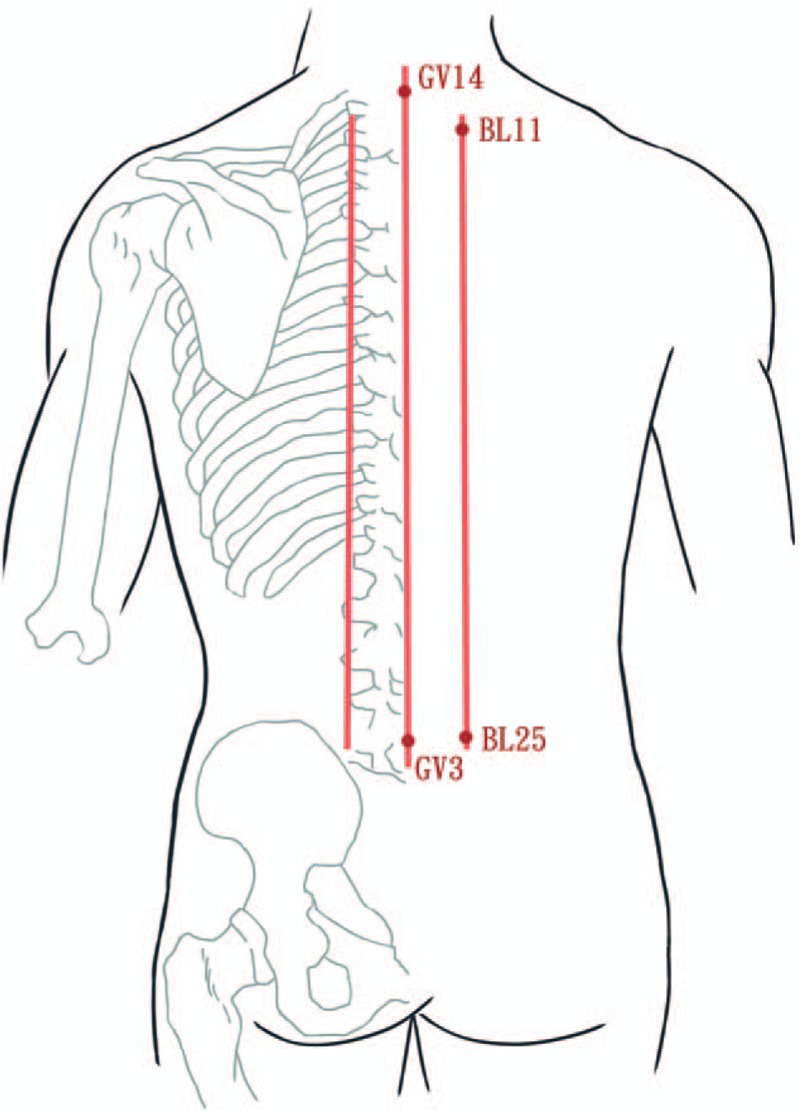
Meridians used for heroin addiction: Bladder meridian (BL11-BL25) and Governor Vessel (GV3-GV14).

**Figure 3:**
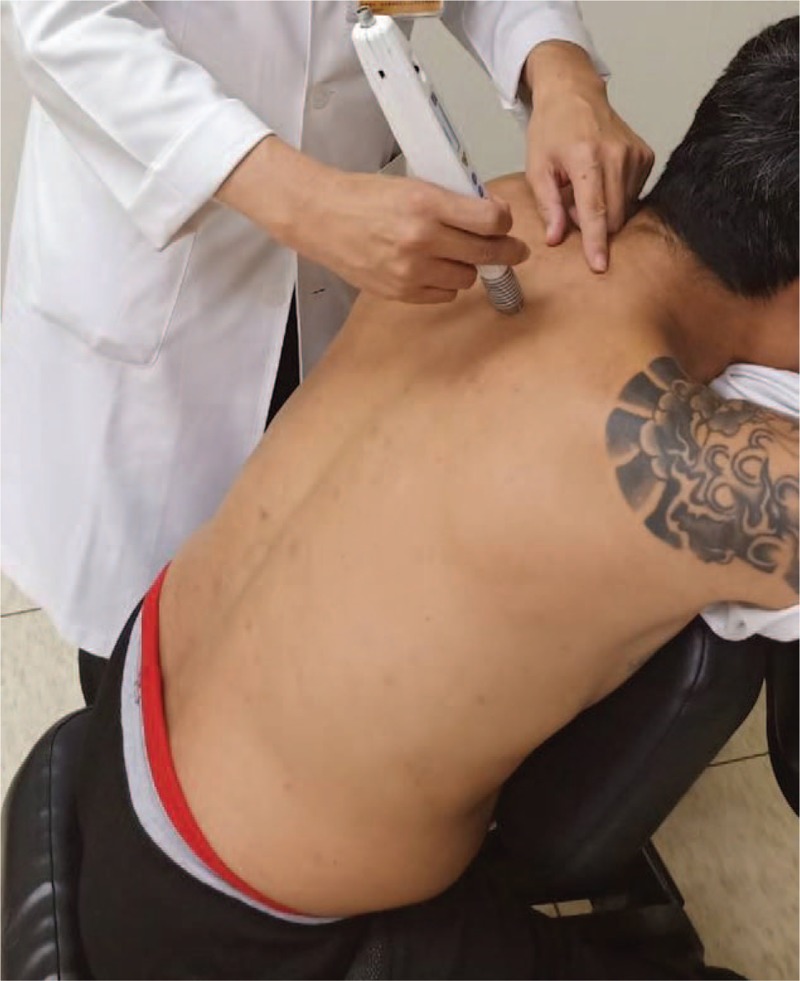
Laser meridian massage performed using the LaserPen device at the Governor Vessel.

The study participants will receive 12 sessions of laser meridian massage three times weekly for 4 weeks using a gallium aluminum arsenide LaserPen (maximal power, 150 mW; wavelength, 810 nm; area of probe, 0.5 cm^2^; power density, 300 mW/cm^2^; pulsed wave; and frequencies [Bl, 667 Hz; B4, 4796 Hz]; RJ-Laser, Reimers & Janssen GmbH, Waldkirch, Germany). The laser treatment will be applied to the back, including the Bladder meridian (BL11–BL25) and Governor Vessel (GV3–GV14; Figs. 2 and 3) for 15 minutes, to deliver a total treatment dose of 67.5 J/cm^2^. The laser treatment will be applied by the same experienced physician who has adequate training and is a licensed Chinese medicine practitioner in Taiwan. The physician and patients are required to wear protective goggles to inhibit visual perception during laser meridian massage.

### 2.6 Outcome measurements

Outcome measures will include subjective reporting of heroin use and quality of life and objective urinary morphine levels. The primary outcomes are urinary morphine levels and self-reported times or days of heroin use during the previous week before and after 2 and 4 weeks of treatment. The secondary outcomes are self-reported visual analogue scale (VAS) scores for heroin craving/refusal to use heroin (0–10 points) in the previous week before and after 2 and 4 weeks of treatment and quality of life using the Short Form-12v2 (SF-12v2) before and after 4 weeks of treatment. The participant's pulse diagnosis and heart rate variability after one single treatment session are also being recorded.

A VAS score of 0 for heroin craving indicates no heroin craving and a score of 10 indicates the strongest possible heroin craving. A VAS score of 0 for refusal to use heroin indicates no refusal and a score of 10 indicates total refusal. The SF-12v2 Health Survey is a multipurpose, short-form health instrument containing 12 questions that yields an 8-scale profile of functional health and well-being (Physical Functioning [PF], Role-Physical [RP], Bodily Pain [BP], General Health [GH], Vitality [VT], Social Functioning [SF], Role-Emotional [RE], Mental Health [MH]), as well as two psychometrically-based physical and mental health summary measures and a preference-based health utility index. The PRO CoRE, which is part of the Smart Measurement System suite of products and Optum's upgrade to the QualityMetric Health Outcomes scoring software, will be used to score the SF-12v2 Health Survey.

The reasons for patients not completing their follow-up visits or dropping out of the study, such as adverse events/intercurrent illnesses, suboptimal response to therapy, failure to return for follow-up, failure to meet the selection criteria at entry, other protocol violations, and refusal to receive treatment, are recorded, as are potential adverse events.

### 2.7 Statistical analysis

The data will be presented as the mean ± standard deviation. The independent *t* test and chi-square test will be used to evaluate and compare the baseline patient characteristics between the experimental and control groups. The independent *t* test and McNemar test will be used to compare the differences between 2 groups. Repeated-measures analysis of variance, generalized estimating equations, and the paired *t* test will be used for comparisons between the 2 study groups. All analyses will be performed using SPSS for Windows, version 22 (Statistics 22, IBM Corp., Armonk, NY). A *P* value < .05 will be considered statistically significant.

### 2.8 Data monitoring

A data monitoring committee (DMC) is not needed because laser meridian massage is a routine and noninvasive intervention.

## 3 Discussion

According to the 2019 report of the United Nations Office on Drug and Crime, the global prevalence of opiate (heroin and opium) use was estimated to be 0.6% in the population aged 15 to 64 years in 2017 (29.2 million); furthermore, the number of past-year opiate users globally was 50% higher than the previously estimated 19.4 million in 2016.^[14]^ The prevalence of heroin use was estimated at 0.23% in the population aged 12 to 64 years in 2014 (42,428, all adults) in Taiwan.^[15]^ Addiction to opiates such as heroin is of great importance because it has such serious sequelae, including high mortality and crime rates. Moreover, impaired cortical plasticity has been detected in heroin addicts, which might account for the addiction scores in these individuals (e.g., craving, relapse) from the perspective of psychomotor stimulant theory or the spiral theory of addiction.^[16]^ Treatment of OUD includes a set of pharmacologic and psychosocial interventions aimed at reducing or ceasing opioid use, preventing future harms associated with opioid use, and improving quality of life and well-being.^[3]^ Pharmacologic strategies have not been successful in management of withdrawal symptoms in heroin addicts; therefore, it is time to investigate the potential benefits of complementary and alternative medicine, which has a long history in the treatment of disease and few side effects.^[17]^

Acupuncture can exert an antinociceptive effect by accelerating the production and release of opioid peptides in the central nervous system. The antinociceptive effect is frequency-dependent, with low-frequency (2-Hz) stimulation accelerating production of endorphin and encephalin and high-frequency (100-Hz) stimulation upregulating the dynorphin level.^[18,19]^ High-frequency (100-Hz) electroacupuncture is effective in reducing withdrawal symptoms, and there is a suggestion that dynorphin suppresses symptoms of withdrawal at the spinal level. The effects of 100-Hz electroacupuncture may include activation of brain-derived neurotrophic factor in the ventral tegmental area, while multiple 100-Hz stimulation may have a cumulative effect related to acceleration of dynorphin synthesis and downregulation of cAMP response element-binding protein.^[20]^ Furthermore, electroacupuncture could effectively alleviate symptoms of opioid craving and depression, and transcutaneous electrical stimulation of acupoints may help to improve symptoms of insomnia and anxiety.^[21]^

In comparison with traditional acupuncture for obtaining qi, laser acupuncture is not related to somatosensation but has the advantage of being noninvasive and aseptic. Furthermore, no heat is generated during the procedure, making laser acupuncture painless and safe.^[22]^ Laser acupuncture integrates the positive effects of both acupuncture and low-level laser therapy.^[12]^ Laser meridian massage is the application of laser acupuncture. Selection of the meridians for detoxification has been extensive. The meridians that include most acupoints are the Governor Vessel (23.5%) and Bladder meridian (14.7%).^[23]^ Therefore, laser meridian massage is expected to be an effective treatment for patients with OUD on methadone maintenance treatment.

## Acknowledgments

The authors thank the Biostatistics Center at Kaohsiung Chang Gung Memorial Hospital for developing the statistical analysis protocol that will be used in this research.

## Author contributions

**Conceptualization:** Wen-Long Hu, Meng-Chang Tsai.

**Data curation:** Wen-Long Hu.

**Funding acquisition:** Wen-Long Hu.

**Investigation:** Wen-Long Hu, Meng-Chang Tsai, Chun-En Kuo, Chun-Ting Liu, Szu-Ying Wu, Tzu-Chan Wu.

**Methodology:** Wen-Long Hu, Meng-Chang Tsai.

**Project administration:** Wen-Long Hu, Meng-Chang Tsai, Chun-En Kuo, Chun-Ting Liu, Szu-Ying Wu, Tzu-Chan Wu.

**Resources:** Wen-Long Hu, Meng-Chang Tsai.

**Supervision:** Wen-Long Hu, Yu-Chiang Hung.

**Validation:** Wen-Long Hu.

**Visualization:** Wen-Long Hu, Yu-Chiang Hung.

**Writing – original draft:** Wen-Long Hu.

**Writing – review & editing:** Meng-Chang Tsai, Yu-Chiang Hung.

Wen-Long Hu orcid: 0000-0001-9549-6202.
